# Whole-genome comparative analysis of virulence genes unveils similarities and differences between endophytes and other symbiotic bacteria

**DOI:** 10.3389/fmicb.2015.00419

**Published:** 2015-05-26

**Authors:** Sebastiàn Lòpez-Fernàndez, Paolo Sonego, Marco Moretto, Michael Pancher, Kristof Engelen, Ilaria Pertot, Andrea Campisano

**Affiliations:** Research and Innovation Center, Fondazione Edmund MachTrento, Italy

**Keywords:** comparative genomics, virulence, core genome, endophyte, synteny, niche occupation

## Abstract

Plant pathogens and endophytes co-exist and often interact with the host plant and within its microbial community. The outcome of these interactions may lead to healthy plants through beneficial interactions, or to disease through the inducible production of molecules known as virulence factors. Unravelling the role of virulence in endophytes may crucially improve our understanding of host-associated microbial communities and their correlation with host health. Virulence is the outcome of a complex network of interactions, and drawing the line between pathogens and endophytes has proven to be conflictive, as strain-level differences in niche overlapping, ecological interactions, state of the host's immune system and environmental factors are seldom taken into account. Defining genomic differences between endophytes and plant pathogens is decisive for understanding the boundaries between these two groups. Here we describe the major differences at the genomic level between seven grapevine endophytic test bacteria, and 12 reference strains. We describe the virulence factors detected in the genomes of the test group, as compared to endophytic and non-endophytic references, to better understand the distribution of these traits in endophytic genomes. To do this, we adopted a comparative whole-genome approach, encompassing BLAST-based searches through the GUI-based tools Mauve and BRIG as well as calculating the core and accessory genomes of three genera of enterobacteria. We outline divergences in metabolic pathways of these endophytes and reference strains, with the aid of the online platform RAST. We present a summary of the major differences that help in the drawing of the boundaries between harmless and harmful bacteria, in the spirit of contributing to a microbiological definition of endophyte.

## Introduction

After the breakthrough of Koch's molecular postulates (Falkow, 2004), bacteriology has been challenged by a new definition of “pathogen.” The fact that horizontal gene acquisition may confer virulence traits to harmless bacteria (Charpentier and Courvalin, 1999; Rosas-Magallanes et al., 2006; Kelly et al., 2009) is changing the dogma on the separation between beneficial or detrimental microorganisms. The interaction in microbial communities can also trigger the disease process (Shankar et al., 2014) and recently a shift of the paradigm from “pathogens” to “pathobiome” was proposed (Vayssier-Taussat et al., 2014). This is especially true for those microorganisms inhabiting complex communities in the gastrointestinal tract of mammals (Britton and Young, 2012), insect guts (Cariveau et al., 2014), in soils and in the phyllosphere, rhizosphere, and endosphere of plants (Troxler et al., 1997; Christensen et al., 1998). Recent studies have cleared that imbalances in host-associated microbial populations, in signaling within bacterial communities and in the immune state of the host play a crucial role in pathogenicity and in disease development (Monack et al., 2004; Parker and Sperandio, 2009; Cardenas et al., 2012).

Plant-borne bacteria can live on the surface or inside their hosts, establishing bonds at different levels, ranging from a loose, free-living lifestyle in the vicinity of the host, to a tight association inside tissues (Little et al., 2008). In the latter case they can live intercellularly or intracellularly without causing any apparent damage or disruption to the plant's homeostasis. Bacteria with this lifestyle are defined as endophytes (Reinhold-Hurek and Hurek, 2011). An endophytic lifestyle benefits a microorganism by providing shelter, facilitating access to carbon sources and increasing its overall fitness. In turn, endophytes may improve plant health and competitiveness by synthesizing molecules involved in plant protection against pests and pathogens (Clarke et al., 2006), nitrogen fixation (Hurek et al., 2002), and induction or enhancement of plant defense mechanisms (Iniguez et al., 2005; Bordiec et al., 2011).

In spite of the experimental evidence showing that many endophytes are beneficial to plants, so far scientists have not succeeded in singling out specific traits that could help categorize bacteria as endophytic organisms. On the other hand, for plant and animal pathogens, genes, and gene families associated with virulent phenotypes have been identified in the past. Several examples exist, including the locus of enterocyte effacement (LEE) in *E. coli* that produces the enterohemorragic phenotype on the gastrointestinal tract (Elliott et al., 2000), the *cag* pathogenicity island in *Helicobacter pylori* responsible for the initiation of the inflammatory response in bacterially-induced gastric ulcers (Backert et al., 2004), or the *hrp* pathogenicity island in the plant pathogenic *Erwinia amylovora*, that encodes a type III secretion system enabling the bacterium to infect the host, ultimately leading to the blighting of tissues and development of disease (Oh et al., 2005).

While endophytes might possess a genetic weaponry similar to that of virulent microorganisms, its expression and regulatory mechanisms are different (Xu et al., 2014), and the coordination of activities and cell to cell communication in the community greatly explains these differences between mutualistic and pathogenic bacteria. This makes the boundaries between the two lifestyles diffuse (Clay and Schardl, 2002; Schulz and Boyle, 2005; Zuccaro et al., 2011; Dubois et al., 2012). For example, the construction of mutants unable to synthesize type IV pili in *Xyllela fastidiosa*, a well-known plant pathogen, showed the impairment for its basipetal translocation in grapevine and a diminished colonization rate, making it more similar to a symbiont (Meng et al., 2005). The analysis of colonization traits and the evaluation of the production of endoglucanases and endopolygalacturonases also showed that the *Burkholderia phytofirmans* strain PsJN indeed utilizes cell-wall degrading enzymes to colonize grapevine tissues (Compant et al., 2005). This phenotype is widespread among pathogens as a means of colonizing plants, thus showing that the definition of endophytes is still *per se* imprecise.

Genome sequencing has become increasingly accessible and the abundance of genomic data is undoubtedly improving our understanding of the evolution of traits involved in mutualism and parasitism (Parkhill et al., 2003; De Maayer et al., 2010; Kahlke et al., 2012). In addition to this, the annotation of genomes and the discovery of biochemical pathways *in silico* has proven a promising tool for bioprospecting in unexplored habitats (Ahmed, 2009). Comparative genomics analyzes several genomes simultaneously to find similarities and differences between them and to study traits that could be further manipulated in the lab by means of genetic engineering (Suen et al., 2010). Genome alignment, synteny plots and core and accessory genome elucidation are basic tools for the genomic study of microorganisms and have proved to be useful in biotechnology (Bentley and Parkhill, 2004; Binnewies et al., 2006).

The increasing knowledge of the composition and organization of chromosomes of endophytes is ultimately providing a body of data large enough to explain complex traits such as plant growth promotion, biocontrol, and symbiosis-specific attributes in different bacteria-plant symbiosystems (Amadou et al., 2008; Fouts et al., 2008; Yan et al., 2008; Tian et al., 2012). To give an example, the analysis of the genome of *B. phytofirmans* PsJN, a grapevine colonizing Gram negative bacterium, has revealed the mechanisms the endophyte uses to colonize the plants. These include bacterial polymer-degrading enzymes, siderophores, and several protein secretion systems (Mitter et al., 2013). These colonization mechanisms are the same used by pathogens to infect plants.

Most of the comparative genomics analyses carried out on endophytes have focused on showing similarities in beneficial traits (Sugawara et al., 2013). Fewer have addressed the main commonalities between endophytes and pathogens. Virulence traits shared by pathogenic bacteria and endophytes could be a milestone for the understanding of adaptation to different hosts and lead to the inception of new tools for the control of detrimental organisms or for improving the performance of beneficial symbionts.

In this work we sequenced the genomes of seven bacterial endophytes isolated from Italian grapevine, belonging to the genera *Enterobacter*, *Erwinia*, and *Pantoea*. We used the sequenced genomes to make comparisons with twelve available genomes of bacteria belonging to the same taxa, but having different lifestyles (endophytic, epiphytic, pathogenic to humans, or pathogenic to plants). Our aim was to determine whether or not the genomes of these endophytes resembled those of organisms engaged in other lifestyles (especially the genomes of pathogens) and whether we could single out specific characteristics of endophytism.

## Materials and methods

### Bacterial strains used in this study

We sequenced the genome of seven bacterial endophytes isolated from Italian *Vitis vinifera* (*Enterobacter ludwigii*. EnVs6, *En*. *ludwigii*. EnVs2, *En. ludwigii*. LecVs2, *Erwinia* sp. ErVv1, *Pantoea vagans*. PaVv1, *P*. *vagans*. PaVv7 *P. vagans* PaVv9) belonging to the family Enterobacteriaceae. The strains were previously characterized in terms of plant growth promotion, antibiotic resistance, exoenzyme production, *N*-acyl homoserine lactone production, and biocontrol activity against plant pathogens (Campisano et al., 2015). Of these seven strains (henceforth referred to as “test strains”), three belong to the genus *Entoerobacter*, three to the genus *Pantoea*, and one to the genus *Erwinia* (Table 1). Twelve available genome sequences of reference strains (four for each genus mentioned above), including either or both human-, plant-pathogenic, endophytic or epiphytic strains, were selected for genome comparison and served as controls for the purpose of this work (*En. cloacae* subsp. *cloacae* ATCC 13047, *En. asburiae* LF7a, *En. aerogenes* KCTC 2190, *En*. sp. 638, *P. agglomerans* 299R, *P. ananatis* LMG 20103, *P. ananatis* PA13, *P. vagans* C9-1, *Er. billingiae* Eb661, *Er. amylovora* ATCC 49946, *Er. pyrifoliae* Ep1-96, *Er. pyrifoliae* Ejp617). We chose four reference genomes for each genus because their sequences are complete and available online. The ecology and properties of these strains (henceforth referred as “reference strains”) have also been studied previously. The identity and genomic characteristics of these strains are summarized in Table 1 and in Supplementary Table ST1.

**Table 1:** **Characteristics of the genomes used in this study**.

**Species**	**Habitat/host**	**Lifestyle**	**Chromosome size (bp)**	**G + C content (%)**	Number of ORF^*^	**References**
*En. cloacae* subsp. *cloacae* ATCC 13047	Human	Pathogenic humans	5,314,588	54.79	5518	Ren et al., 2010
*En. asburiae* LF7a	Human	Pathogenic humans	5,012,130	53.84	4612	Brenner et al., 1986
*En. aerogenes* KCTC 2190	Human	Pathogenic humans	5,280,350	54.8	4912	Shin et al., 2012
*En*. sp. 638	Poplar	Endophytic	4,518,712	52.98	4240	Taghavi et al., 2010
*Er. billingiae* Eb661	Pear tree	Epiphytic	5,100,168	55.2	4587	Kube et al., 2010
*Er. amylovora* ATCC 49946	Apple tree	Pathogenic plant	3,805,874	53.5	3483	Sebaihia et al., 2010
*Er. pyrifoliae* Ep1-96	Pear tree	Pathogenic plant	4,026,322	53.4	3645	Kube et al., 2010
*Er. pyrifoliae* Ejp617	Pear tree	Pathogenic plant	3,909,168	53.64	3873	Park et al., 2011
*P. agglomerans* 299R	Pear tree	Endophytic	4,581,483	54.29	4194	Remus-Emsermann et al., 2013
*P. ananatis* LMG 20103	Eucalyptus tree	Pathogenic plant	4,690,000	53.69	4241	De Maayer et al., 2010
*P. ananatis* PA13	Rice plants	Pathogenic plant	4,586,378	53.66	4372	Choi et al., 2012
*P. vagans* C9-1	Apple tree	Epiphytic	4,025,000	55.09	4619	Smits et al., 2010
*En*. *ludwigii*. EnVs6	Grapevine plants	Endophytic	5,220,112	54.62	4809	This study
*En. ludwigii* EnVs2	Grapevine plants	Endophytic	5,067,900	53.98	4649	This study
*En. ludwigii* LecVs2	Grapevine plants	Endophytic	5,285,925	54.59	4886	This study
*Er*. sp. ErVv1	Grapevine plants	Endophytic	4,719,019	54.6	4207	This study
*P. vagans* PaVv1	Grapevine plants	Endophytic	4,850,774	55.14	4453	This study
*P. vagans* PaVv7	Grapevine plants	Endophytic	4,879,255	55.22	4470	This study
*P. vagans* PaVv9	Grapevine plants	Endophytic	9,754,510	54.65	9466	This study

* *ORF stands for open reading frames*.

### DNA extraction, genome sequencing and assembly

The test strains were grown in nutrient broth (NB; Oxoid, United Kingdom) at 200 r.p.m on a SI600R rotatory incubator (MID SCI, USA) at 30 ± 2°C until an OD_600_ value of 0.8. Bacterial cells were pelleted on an ANNITA PK12 R bench centrifuge (ALC International, Italy) for 5 min at 10,000 r.c.f. The pellet was washed with sterile phosphate buffer saline (PBS) 1× and DNA was extracted using the RBC real genomics DNA extraction kit (RBCBiosciences, China) according to the manufacturer's instructions. DNA concentration was estimated using a Nanodrop 8000 UV-VIS spectrophotometer (Thermo Scientific, Germany). DNA integrity and the absence of RNA contamination were checked by electrophoresis on a 1% agarose gel. Sequencing libraries were constructed using the Nextera DNA Sample Prep Kit (Illumina, Inc., USA) according to the manufacturer's instructions. DNA-seq libraries were pooled at 10-plex level of multiplexing and sequenced paired-end 100 bp on a HiSeq2000 Illumina sequencer at IGA Technology Services (Udine, Italy). Raw images were processed using Illumina Pipeline version 1.8.2.

The assembly of the genomes from test strains was performed as follows. Three of the genomes (EnVs6, ErVv1, and PaVv9) were assembled using the A5 pipeline release 20140401 (Tritt et al., 2012); other four assemblies (PaVv7, EnVs2, LecVs2, and PaVv1) were produced using the SOAPdenovo software, version 2.04 (Luo et al., 2012). The assemblies were done using the two aforementioned pipelines as they provided the optimal number of contigs and N50, after testing different methods. Quality of the sequences was evaluated using the quality assessment tool for genome assemblies (QUAST) to produce metrics of quality using two different assembly pipelines (Alexey et al., 2013).

### Annotation and subsystem analysis

Genomes were annotated using the Prokaryotic Genome Annotation System (PROKKA) (Seemann, 2014). To eliminate the bias of different annotation systems employed, test and reference genomes were all submitted to the online platform RAST (Rapid Annotation using Subsystem Technology) version 2.0.

We chose six RAST subsystems central to our analysis (cell wall and capsule, iron acquisition and metabolism, chemotaxis, phages and mobilome, regulation and cell signaling, virulence and disease) to build a presence/absence map through a series of Perl® scripts (Wall et al., 2000), which summarize all pairwise comparisons into a unique list, including all genes assigned to the chosen subsystems. A list of genes common to all compared genomes was compiled using a custom script in R version 3.0.2 (R Core Team, 2013).

### Phylogeny

We retrieved the sequences of the test strains' 16S rDNA genes from RAST and confirmed that they were identical to those previously deposited in GenBank (Campisano et al., 2015). We then used the 16S rDNA sequences downloaded from RAST to perform a blastn search against the NCBI database (Wheeler et al., 2007), and aligned them with the closest sequences from the database using the clustalW algorithm implemented in the software bioedit version 7.2.5 (Hall, 1999).

We reconstructed the phylogeny using the tree-building algorithm Neighbour-Joining with the Jukes-Cantor distance estimator implemented in MEGA6 version 6.0.6 (Tamura et al., 2013). We assigned the phylogeny, when possible, to the species level.

### Whole-genome comparison

We used several methods for whole-genome comparison to identify similarities and differences between each test strain's genome and reference genome in the same genus. First we retrieved the assembled genomes from RAST and aligned them against one reference genome for each genus using MAUVE version 2.3.1 (Darling et al., 2004). Following this step we performed a multiple whole–genome alignment using the progressive alignment algorithm implemented in MAUVE. We used the output of this alignment to check for rearrangements in each genome.

Syntheny plots were constructed as had been done previously (Husemann and Stoye, 2010) by aligning regions of the test and reference genomes that differed by no more than 8% and shared at least 44 overlapping 11mers no more distant from each other than 64 nucleotides. All regions were aligned and displayed in r2cat (Husemann and Stoye, 2010). We drew several plots to check for syntheny against reference strains with different degrees of relatedness, for each of the three genera under study.

We constructed a circular genomic map for each genome using the BLAST Ring Image Generator (BRIG, version 0.95; Alikhan et al., 2011). Each circular genomic map was drawn using the genome of one reference strain (henceforth referred to as “alignment reference genome”) on a local BLAST + basis, with standard parameters (50% lower – 70% upper cut-off for identity and *E*-value of 10). For the genus *Enterobacter* we used the fully sequenced genome of the poplar endophyte *Enterobacter* sp. 638 as a reference to align all genomes under this genus. For the genus *Erwinia* we used the sequenced genome of *Er. amylovora* ATCC 49946. For *Pantoea* we used the genome sequence of the biocontrol agent *P. vagans* C9-1. The ring color gradients correspond to varying degrees of identity of BLAST matches. Circular genomic maps also include information on GC skew and GC content.

### Clustering of orthologous families

In order to depict the core and accessory genome in each genus, we performed a reciprocal best hit search using the OrthoMCL software release five (Li et al., 2003). For this we downloaded the predicted coding sequences (CDS) of the reference and the test strains and performed a blastp search against each other with an *E*-value cut-off of 10^−5^ and a sequence coverage higher than 50%, as reported previously (Li et al., 2013). We used a series of built-in scripts to (i) parse, (ii) upload to the MySQL relational database, (iii) perform a reciprocal best hit analysis to form pairs of sequences, and to (iv) normalize the *E*-values for all the pairs formed. Normalization of *E*-values was done by by averaginf all recent orthologs (in paralogs) and dividing each pair of orthologs by the average Finally, using the Markov Cluster Algorithm (MCA) program, the sequences were distributed in orthologous families using 1.5 as the inflation value. We calculated the pangenome size as the sum of the CDS in all the genomes and the accessory genome size (within each genus and for each species) as the difference between the pangenome and the core genome. We assigned functions to all orthologous families and filtered virulence functions using a set of Perl® scripts. We also used a list of keywords to query for virulence functions in the orthologous families and to calculate the number of matches of those functions, using custom bash commands.

### Secretion system analysis

The Lawrence Livermore national laboratory virulence database (MvirDB; Zhou et al., 2007) and the virulence factors of pathogenic bacteria database (VFDB; Chen et al., 2012) contain hundreds of gene annotations related to virulence determinants of bacteria and have been curated since 2007. We used these resources to download datasets of virulence genes (either validated or predicted). We chose a set of bacterial secretion systems (ranging from type I to type VII) to locate secretion system genes in the genomes of the test strains. The matching results were drawn in a presence/absence map.

### CRISPRs and phage presence

To evaluate the presence of CRISPRs (Clustered Regularly Interspaced Short Palindromic Repeats) we analyzed the assembled genomes of the seven test strains using the CRISPR Recognition Tool CRT (Bland et al., 2007). Also we search for putative phage sequences in the test strains in the online tool PHAST (Zhou et al., 2011).

## Results

### Sequencing and assembly of endophytic genomes

The characteristics of the genomes sequenced in this study, including length, GC content and amount of transfer, and ribosomal RNA genes are summarized in Table 1 and in Supplementary Table ST1. The sequencing of the 16S rDNA revealed that the test strains under the genus *Enterobacter* are closely related to *En. ludwigii*; strain ErVv1 under the genus *Erwinia* is related to *Er. amylovora* and *Er. tasmaniensis*, while the *Pantoea* test strains are closely related to *P. vagans* (Supplementary Figure SF1). The sequencing data is deposited in the EMBL-EBI repository (https://www.ebi.ac.uk/ena) with accession numbers PRJEB8251 (EnVs6); PRJEB8253 (EnVs2); PRJEB8254 (LecVs2); PRJEB8255 (PaVv1); PRJEB8258 (PaVv7); PRJEB8259 (PaVv9); and PRJEB8284 (ErVv1).

To optimize the assembly procedure, we compared the performance of two different pipelines (A5 and SOAPdenovo) through the online tool QUAST (Supplementary Table ST2). The realignments of the reads showed consistently good results with high realignment percentages and the distribution of the insert sizes within the limits of the suggested values from the sequencing provider (~600/800 bp). The Log Aaverage Probability (LAP) scores (Ghodsi et al., 2013) from the different assemblies for the same organism were also very close, showing that both pipelines produced comparable results. We used this scores and the N50 values to choose which assembly pipeline to use. For strain LecVs2, pipeline A5 did not produce any results. Thus, we used the Velvet pipeline (Zerbino and Birney, 2008) to assemble the genome and compared it with the SOAPdenovo assember. Quality analysis showed that differences between the two pipelines were minimal as presented in Supplementary Table ST2. Scripts to build up the assemblies are deposited and publicly available at the github online public repository (at the address https://github.com/pochotustra/genomics_endophytes.git as Supplementary Material SM1-4

### Comparison of genome structure between endophytic and non-endophytic genomes

**Figure 1:**
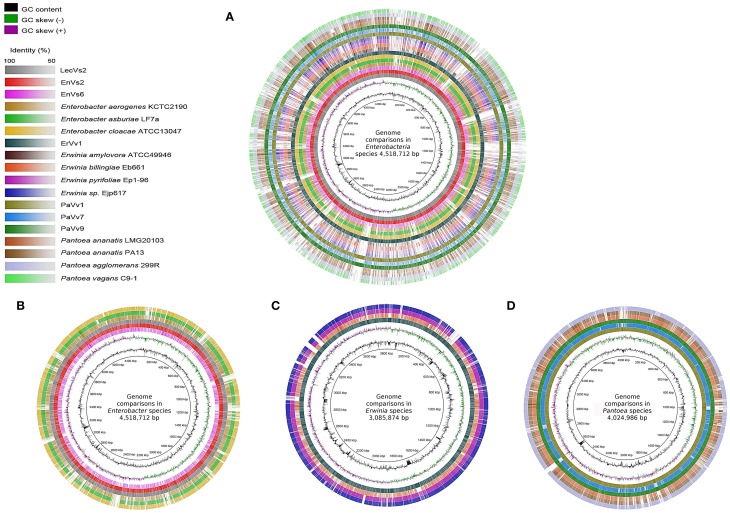
**Whole-genome comparisons in three genera of Enteobacteria**. The color intensity in each ring represents the BLAST match identity. **(A)** whole-genome comparison of all the strains considered in this work. **(B)** Whole-genome comparisons in *Enterobacter*, from outer to inner ring: *En. cloacae* subsp. *cloacae* ATCC 13047, *En. asburiae* LF7a, *En. aerogenes* KCTC 2190, *En. ludwigii* LecVs2, *En. ludwigii* EnVs2, *En. ludwigii* EnVs6; reference genome: *Enterobacter* sp. 638**. (C)** Whole-genome comparisons in Erwinia, from outer to inner ring: *Er*. ErVv1, *Er. pyrifoliae* Ejp617, *Er. pyrifoliae* Ep1-96, *Er. billingiae* Eb661; reference genome: *Er. amylovora* ATCC 49946. **(D)** whole-genome comparisons in *Pantoea*, from outer to inner ring: *P. agglomerans* 299R, *P. ananatis* PA13, *P. ananatis* LMG 20103, *P. vagans* PaVv9, *P. vagans* PaVv7, *P. vagans* PaVv1; reference genome: *P vagans* C9-1.

A visual inspection of the circular alignment of genomes under the genus *Enterobacter* (Figures 1A, B) highlights that two of the test genomes (EnVs2 and LecVs2) are similar to the alignment reference genome of endophytic strain 638, while the test strain EnVs6 is similar to the reference genomes of human pathogens (ATCC 13047, LF7a, and KCTC 2190). The region between 1–800 kbp is well conserved in all three test isolates (EnVs6, EnVs2, and LecVs2). The rest of the chromosome is more variable, with regions where the identity between EnVs2 and LecVs2 reaches up to a 70% while the test strain EnVs6 aligns better with the genomes of pathogens (ATCC 13047, LF7a, and KCTC 2190). A positive to negative GC skew at position 1 corresponds to the origin of replication and the switch at 2200 kbp may account for the replication terminus. Regions at 2800–2880 and 3120–3200 kbp have higher content of GC. Several regions in the genome of test strains (EnVs2 and LecVs2) are syntenic with the genome of the alignment reference genome of endophytic strain 638. For strain EnVs6 however this synteny is less conspicuous as it is made evident in the alignment plot (Supplementary Figures SF2A–C). Regions between 240–280 kbp and between 4040–4080 kbp in the test strains (EnVs2, EnVs6, and LecVs2) show an identity higher than 70% to the alignment reference genome of strain 638. In these regions we located genes related to the thiamin biosynthesis (the thiamin phosphate pyrophosporilase and the thiamin biosynthesis gene *thiC*) and several genes of the *yjb* operon that regulate the synthesis of a stress-induced exopolysaccharide in *E. coli* (Ionescu et al., 2008). We also found that more than 10 regions in the genome of strain 638 and of the test strains (EnVs2 and LecVs2) are absent in the pathogenic reference strains and in one test strain (ATCC 13047, LF7a, KCTC 2190, and EnVs6). These missing regions contain several enzymes involved in nitrogen assimilation including genes coding for a 2,3,4,5-tetrahydropyridine-2,6-dicarboxylate *N*-succinyltransferase, a uridylyltransferase and several enzymes for the synthesis of the core carbohydrate 3-deoxy-D-manno-octulosonic acid (KDO).

In the genus *Erwinia* a positive to negative GC skew at position 1 in Figure 1C corresponds to the origin of replication and a positive to negative GC switch at approximately 1920 kbp corresponds to the replication terminus in all the genomes compared. We found seven positions with higher GC contents in regions 440–480, 600–960, 1280–1320, 1640–1720, 2200–2640, 2880–2920, and 3340–3400 kbp. These regions contain several virulence factors including *vgrG, virB* and several *imp* all genes related to the type VI secretion system (Filloux et al., 2008). Also, several genes of the biotin biosynthesis pathway and a few DNA repair genes like the methylated DNA protein cysteine methyltransferase are found in these regions, according to the location on the genome of the alignment reference genome ATCC 49946.

A qualitative interpretation of the plots suggests that the genome of strain ErVv1 is similar to that of the alignment reference genome ATCC 49946 and they share several regions that are not present in the other genomes compared. Conversely, the reference strains Ep1-96, Eb661, and Ejp617 are more similar to each other. The more evident gaps highlighting the missing regions are visible at positions 40–60, 440–530, 2400–2440, 2920–2960, 3120–3160, 3220–3240, and 3360–3400 kbp (Figure 1C). Synteny plots constructed for the test strain genomes under taxa *Erwinia* show a high degree of homology with the alignment reference genome. The number of insertions or deletions is low, which is confirmed by the continuity in the plots. For strain ErVv1 the number of discontinuities is higher (6 regions of the genome being separated by indels; Supplementary Figure SF2D).

In Figure 1D we compare test and reference strains of the *Pantoea* group. The test strains PaVv1 and PaVv9 are similar to the C9-1 strain used as alignment reference genome, while the test strain PaVv7 is very similar to the genomes of pathogens PA13 and LMG20103. We found regions with higher GC content at positions 960–1120, 1800–2200, 2560–2600, and 2840–2920 kbp. The GC switches at 1440 kbp (positive to negative) and at 3480 kbp (negative to positive) may represent the origin of replication and replication terminus, respectively (Figure 1D). The sequence identity between the test strains (PaVv1, PaVv7, and PaVv9) and the alignment reference genome C9-1 is high throughout the alignment and can reach peaks of up to 100%. This is more evident in the regions between 200–1700 and 2400–4000 kbp. Several regions shared between the alignment reference genome and the test strains are instead absent in the genomes of pathogens PA13 and LMG20103. The genes differentially present in these regions are involved in cysteine metabolism, isoprenoid biosynthesis and in the transport and metabolism of D-glucarate (ascorbic acid biosynthesis).

Synteny plots made for *Pantoea* show homology of test strains to the alignment reference genome C9-1. We located two exceptional re-arrangements (indels) in strains PaVv9 and PaVv1 that are not present in strain PaVv7 (Supplementary Figures SF2E–G).

### Commonalities and differences in the core and accessory genome of enterobacterial strains

#### Genus Enterobacter

**Figure 2:**
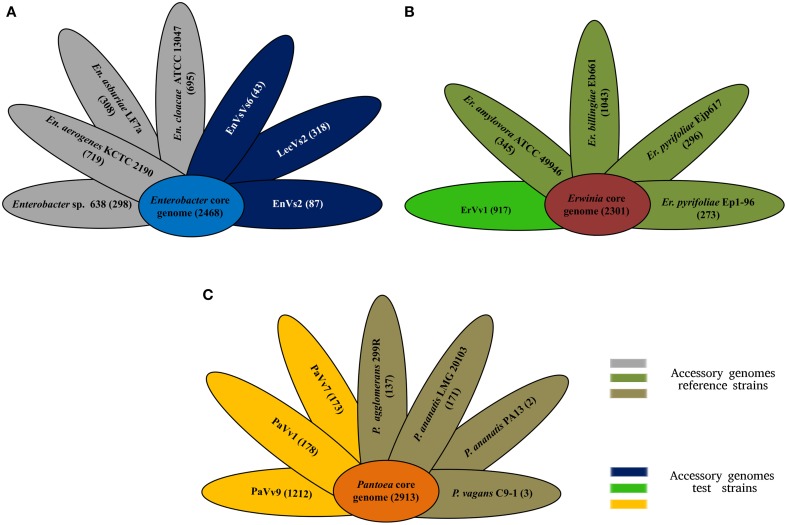
**Core and accessory genome size**. **(A)** Core (sky blue) and accessory genome size of reference (gray petals) and test (deep blue) strains of the genus *Enterobacter*. (**B)** Core (terracotta) and accessory genomes of reference (olive petals) and test (bright green petals) strains of the genus *Erwinia*. **(C)** Core (orange) and accessory genomes of reference (brown petals) and test (yellow petals) strains of the genus *Pantoea*. Numbers indicate genes counts.

**Figure 3:**
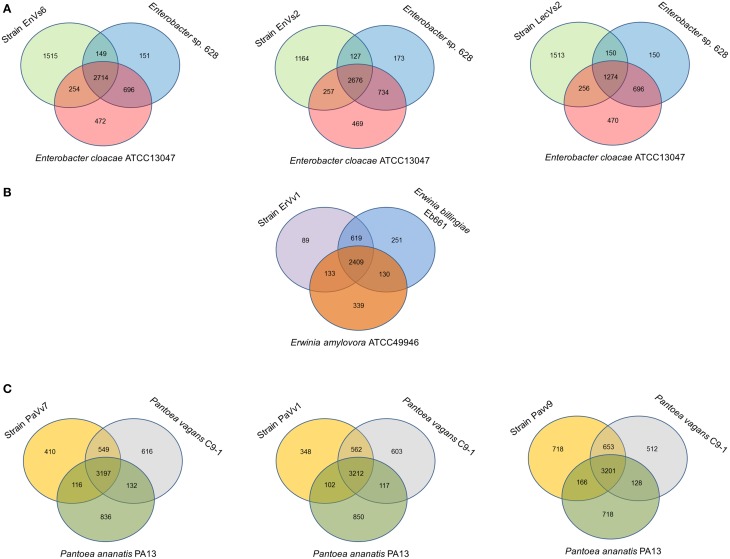
**Venn diagrams showing shared and unique genes in *Enterobacteriaceae***. All comparisons are made between the test strains (left circle) with an epiphyte or endophyte (right circle) and a pathogen (lower circle). **(A)** Comparison of strains EnVs6, EnVs2, and LecVs2 with the endophyte *Enterobacter*. sp. 638 and the human pathogen *En. cloacae* ATCC 13047**; (B)** comparison of strain ErVv1 with the epiphyte *Er. billingiae* strain Eb661 and the plant pathogen *Er*. *amylovora* ATCC 49946; **(C)** comparison of strains PaVv7, PaVv1, and PaVv9 with the epiphyte *P. vagans* C9-1 and the plant pathogen *P. ananatis* PA13. Numbers inside the circles, indicate the genes shared among genomes.

Our analysis shows that the core genome in this taxon comprises 2468 orthologous genes that correspond to 53% of the pangenome (Figure 2A and Supplementary Figure SF3). Venn diagrams show genes exclusively shared between test strains and either of the reference genomes. In strains EnVs6, EnVs2, and LecVs2, the number of genes shared exclusively with the endophytic reference strain 638 was 149, 127, and 150, respectively. The number of genes shared exclusively with the pathogenic reference strain (ATCC 13047) was 254, 257, and 256, respectively (Figure 3A).

**Figure 4:**
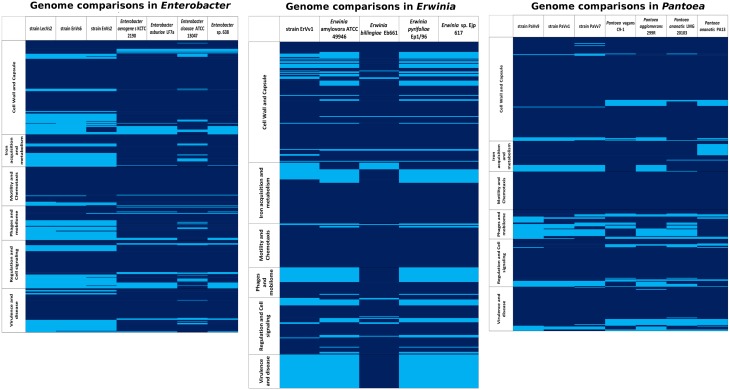
**Presence/absence map based on six functional gene categories (from RAST)**. Rows: within categories, each line represents a gene; deep blue, gene presence; sky blue, gene absence. Columns: each column represents an analyzed genome in the genus *Enterobacter*, *Erwinia*, or *Pantoea*.

In the genus *Enterobacter*, the abundance of genes in each category (see Supplementary Table ST3 for a list of categories used) was similar in all genomes (Figure 4). The cell signaling and two-component system genes are present in all of the strains analyzed. In these systems, transcriptional regulators belonging to the LysR and GntR families (Fujita and Fujita, 1987; Maddocks and Oyston, 2008) are the most important. We found a conserved set of about 40 genes devoted to the synthesis of cell wall and capsule that are shared among all *Enterobacter* genomes under analysis. Among these, we highlight the presence of *rlpB*, *rcsF*, *lptA*, *lptC* and the organic solvent tolerance protein *lptD*a set of genes involed in LSP biosynthesis (McCandlish and Silhavy, 2007) and the oxidoreductases *mviM* and *mviN*, necessary for murein synthesis (Inoue et al., 2008). We found an average of 51.8 flagellar genes across the *Enterobacter* genomes with a maximum in the reference pathogenic strain ATCC 13047. A set of genes that belong to the *che* operon for chemotaxis signaling and to the twitching motility apparatus are also present in the core genome. The number of genes related to pathogenicity mechanisms was slightly higher in the genomes of the test strains LecVs2 and EnVs6 and in the genome of the reference genome ATCC 13047. Among these genes we emphasize the presence of a virulence sensor related to the *bvgS* sensor kinase and of *arnC* belonging to the polymixin resistance group. We report the presence of a type 1 secretion system agglutinin of the RTX family (Linhartová et al., 2010) and members of a tripartite multidrug resistance system. Some of the genes in this category are also related to the type II secretion systems and genes for the biogenesis of type IV pilus. The categories with the lowest number of genes were phages and *quorum sensing* in which the highest number of genes among the test strains was in the genome of EnVs6 (Figure 4) Several protein coding sequences for phage capsid and phage associated enzymes (terminases and integrases) are shared among all strains. In the *quorum sensing* category we detected the presence of the *sdiA* gene of the orphan *quorum sensing* communication circuit from *E. coli* (Kanamaru et al., 2000). We also located the a *N*-acyl homoserine lactone synthase in all genomes. No AI-2 dependent *quorum sensing* systems were detected in the test strains. We found no variation in the content of siderophore related genes (average 52,14 genes). However, the test strains (EnVs6, EnVs2, and LecVs2) contain a higher number of genes for this category as compared to all the reference strains (Figure 4) We found the genes for enterobactin synthesis and some of the genes for hemin metabolism. Also, exo- and endoenzymes are present in the core genome of the *Enterobacter* including the phospholipase A and two hemolysins.

#### *Genus* Erwinia

In the genus *Erwinia*, the core genome is constituted by 2301 gene clusters that correspond to the 60% of the pangenome (Figure 2B and Supplementary Figure SF3). Venn diagrams in Figure 3B show genes exclusively shared between test strains either the endophytic reference genomes or the pathogenic reference genomes. We found that strain ErVv1 shares 133 genes exclusively with the apple tree pathogen ATCC 49946 and shares 619 genes exclusively with the epiphyte Eb661.

The core genome of the genus *Erwinia* defined in this study is populated with cell signaling functions including two component systems sensitive to nitrates, copper and osmolarity (Figure 4). We located also two transcriptional factors belonging to the Rrf2 family involved in the metabolism of cysteine (Shepard et al., 2011) and a cyclic AMP regulator of the *crp/fnr* family (Shimada et al., 2011). Among the most important gene functions for cell wall is the regulator in colanic acid synthesis that confers a mucoid phenotype in other taxa. The flagellar machinery is present in all the strains analyzed. As pathogenicity mechanisms we detected multidrug efflux transporters as well as heavy metal detoxification genes including the *arcB* gene (Iuchi et al., 1990) and the cation efflux pump *fieF* (Munkelt et al., 2004); it also bears well-known antibiotic inactivating proteins, including the *ampG* beta lactam activation protein (Lindquist et al., 1993) and both *mdtK* and the multidrug resistance protein D *emrD* (Lomovskaya and Lewis, 1992). Several phage elements and the *sdiA* gene from the *quorum sensing* circuit along with a *N*-acyl homoserine lactone synthase and a homoserine lactone transporter are conserved in all species analyzed. The core genome contains also general mechanisms for iron acquisition that comprise 36 different functions. In the exoenzyme category we found two cellulose synthesis genes, one phospholipase A and a cryptic hemolysin regulator.

#### *Genus* Pantoea

In the genus *Pantoea* we found 2913 orthologous families. The core genome corresponds to 60% of the pangenome (Figure 2C and Supplementary Figure SF3). Venn diagrams show that strain PaVv7 shares 549 genes exclusively with the symbiont C9-1 while test strains PaVv1 and PaVv9 share 562 and 653, respectively. One-hundred and sixteen genes are shared exclusively between the rice pathogen PA13 and the test strain PaVv7 while test strains PaVv1 and PaVv9 share 102 and 166 genes, respectively (Figure 3C).

The cell signaling mechanisms in the core genome of *Pantoea* include the diguanylate cyclase mediator of biofilm formation, along with other biofilm-related genes like *rcsB* (Shiba et al., 2006), and one ribose metabolizing gene *rpiR* (Sorensen and Hove-Jensen, 1996). In this category two distinct groups*: one* made of 299R, LMG 20103, PaVv1, and PaVv7 and a second group made of PaVv9, C9-1, and PA13 are well defined (Figure 4) The cell wall and capsule functions form the same two groups, in which the second group has a greater number of genes in the core genome (Figure 4). The functions for these genes are linked to the synthesis of the exopolysaccharide substance amylovoran and of lipopolysaccharide modifications. The presence of a putative cellulose synthesis gene is consistent in all the species. Among the flagellar genes we were able to recognize three different groups: 299R and LMG20103; PaVv1 and PaVv7; PaVv9, C9-1, and PA13. As for pathogenicity mechanisms, we found that the number of genes in strain PaVv9 is the highest among the test strains (282 genes) and similar to the number found in the reference plant pathogen PA13 (290 genes). Also a high number of genes for flagellum assembly (139 genes), exo- and endo-enzymes (117 genes), capsule (141 genes), and phages (37 genes) is present in this test strain PaVv9 as compared to the other test strains and to some of the reference genomes (Figure 4). Inside the core genome, we located the *shlA* gene and several other putative adhesines. We also found the *pilN* gene of pilus biogenesis (Sakai and Komano, 2000) along with the two partner secretion system *tpsA/B* that correspond to a conserved virulence factor for host adhesion and toxicity in bacterial cells (Ur Rahman and Van Ulsen, 2013). Finally, we provide evidence for the presence of the phenazine synthesis gene *phzF* (Parsons et al., 2004). Some of the virulence-related gene products are transmembrane transporters including members of the major facilitator superfamily (MFS) and members of the Tol-Pal system involved in membrane integrity and phage acquisition. The core genome of the *Pantoea* is poor in genes related to iron acquisition although it contains important elements belonging to the ABC transporters and to the TonB-dependent pathway. Also in the core genome of the *Pantoea* species analyzed in our study, several exo- and endo-enzymes were detected. Particularly, we were able to track the *bcs* genes involved in cellulose metabolism. Several regulatory proteases make part of the core genome of the genus. We found *hsl*, *clp*, *lon*, and *fts* present in all the genomes analyzed. We also detected a lipase gene LIP-1 and the collagenase *yhbU* that has been implicated in virulence of several animal pathogens. We report also the finding of a LuxI-type coding gene, similar to the *pagI* synthase and a transcriptional activator belonging to the LuxR family that's related to the *N*-3-oxohexanoyl-L-homoserine lactone quorum-sensing transcriptional activator of *P. agglomeran*s (Figure 4).

### Unique gene functions in the different lifestyles

Presence/absence maps based on six RAST categories from annotated genomes of test and reference strains were obtained (Figure 4). In each genus, a list of shared and unique features of virulence-related genes was drawn (Supplementary Table ST4). The genomes of both reference and test strains in all genomes contain most of the main functions for cell wall synthesis and flagellum synthesis which are basic for the niche occupation. They are characterized however, by minor differences in which some genes are unique for test strains or for reference strains, respectively.

In the genus *Enterobacter* some genes involved in the modification of lipopolysaccharide as well as genes related to the glicocalix synthesis and modification are lacking in the test strains (Supplementary Table ST5). Genes related to the use of sialic acid are present only in the test and absent in the reference genomes. We also found that genes involved in the metabolism hemin and synthesis of aerobactin are absent in the test and present in the reference genomes. We detected variable content of flagellar genes since test strains possess one component of the flagellum apparatus uniquely present and lack one gene of the *che* group.

In the genus *Erwinia* we highlight four genes that are absent in the genomes of the reference strains with unrelated functions: antibiotic resistance, detoxification of xenobiotics and stress response (Supplementary Table ST6).

In the genus *Pantoea* we stress the presence of genes that are unique only in the test strains. These genes are necessary for plasmid stability and for recognition and resistance to exogenous substances. On the other hand, the test strains lack genes for modification of the KDO in the LPS an one sequence coding for structural components of a phage, that are only present in the reference strains (Supplementary Table ST7).

### Other virulence determinants: screening for CRISPRs, phages, and secretion systems

#### CRISPRs

We identified only two CRISPR systems, located in the genome of strain EnVs6 (Table 2). The systems consist of two sets of repeats of approximately 20 nt each. The first set consisting of only three spacers and four repeats and the second system with seven repeats and six spacers. No CRISPRs were identified in the genomes of strains LecVs2, EnVs2, ErVv1, PaVv1, PaVv7, or PaVv9.

**Table 2:** **CRISPRs found in the genome of strain EnVs6**.

**Range**	**Position**	**Repeat**	**Spacer**
CRISPR 1 Range: 556393–556547	556393	CGCCATTCATGGCGACCTT	ATTAATGGCCGCACCCTGCCCCG
	556435	CGCCATTCATGGCGACCTT	CTTCTTACACGCACCCAACNTAATCCGTAGGGT
	556487	CGCCATTCATGGCGACCTT	ATTAATGGCCGCACCCTGCCCCG
	556529	CGCCATTCATGGCGACCTT	
CRISPR 2 Range: 2949702–2949985	2949702	ATGTTCACTGTAATCAGTAAA	ACTTGATGACTTTTCTTTCTCAACGCCTA
	2949752	ACATTCACTGTAATCAGTGAA	AACCTTGTGCTCATCATAGACAA
	2949796	AGATTCACTGTAGTCAGTAGA	TGGTTATCGCGCTTCGATTTA
	2949838	ACGTTCACTGTAATCAGTAAG	TCAAATCTGCAACTTCGACAGA
	2949881	ACATTCACTGTAATCAGTAAA	AGTTATGACCCGGAGAAGA
	2949921	ACGTTCACTGTAATCAGCAAA	GCTTGTTTAGTACTTTGATACGA
	2949965	GCGTTCACTGTAATCAGTAAA	

#### Phages

With RAST we observed the presence of several phage-related proteins including the tail and sheath proteins as well as DNA modifying proteins of phage origin (Supplementary Tables ST5–7), We used PHAST to confirm the presence of phages that we were able to identify with RAST. With PHAST we were able to identify 12 intact phages in the genomes of the test strains EnVs6, LecVs2, EnVs2, PaVv9, PaVv1, PaVv7 (Supplementary Table ST8). The phage sequences belong to different types of phages, and are identical to those present in *Enterobacter* genomes deposited in NCBI. The phages do not seem related to one another.

#### Secretion systems

We detected some of the components for major secretion system in all test strains (Supplementary Table ST9). In all the test strains we detected some of the flagellar components plus the protein *clpV*. In *Enterobacter*, all test strains contain the hemaglutinin/adhesin gene *shlA* (Poole and Braun, 1988). In *Erwinia* we did not find any common secretion system gene among the test and reference strains and in *Pantoea* test strains we found the protein Sfa3 of the type VI secretion system apparatus in *Pseudomonas aeruginosa* (Sana et al., 2013).

## Discussion

In this work we compared the genomes of seven bacterial grapevine endophytes, belonging to three genera of the family Enterobacteriacea using several methods including whole-genome alignments and synteny plots. We selected this taxon because it contains some of the most important human and plant pathogens (*Escherichia coli*, *Salmonella typhimurium*, *Shigella flexneri, Pantoea ananatis*, and *Erwinia amylovora* among several others) but also includes beneficial species. We also calculated the core and accessory genomes and showed that the core genome of three genera of enterobacteria are populated with virulence factors. Further, we provide evidence for ecological genus-level trends in endophytic organisms, reflected in their genomes' organization. We also highlighted a set of genes that are uniquely present in some isolates, that may possibly account for specificity in niche occupation. Finally, using a subsystem categorization of the main virulence traits in annotated genomes, we revealed that endophytes bear a very similar set of virulence traits within their genomes and that the patterns of gene distribution can be comprable to that of pathogens, fulfilling one of the conditions of the pathobiome hypothesis.

Our study clearly shows how, endophytic test genomes are not only similar to reference endophytes but also share several characteristics with pathogenic reference genomes (Figure 1 and Supplementary Figure SF2). At the structural level, We have demonstrated a high degree of synteny between endophytic test and reference strains (Supplementary Figure SF2) but also a high identity percentage at the genome level between endophytes and symbionts with other lifestyles (Figure 1). We hypothesize that these traits reflect the potential of bacterial endophytes to express virulence when associated with their hosts and therefore the similarity of pathogens and endophytes at the genomic level. In *Pantoea* for example, the similarities at the structural level between endophytes and plant pathogens are linked to the absence of regions that contain key enzymes for aminoacid biosynthesis and vitamin production. In *Enterobacter* it is the absence of genes for central metabolism and of some, but not all, genes for exopolysaccharide modification (that are otherwise present in the endophytic reference strain 638) and in *Erwinia* the similarities between test and pathogenic reference genomes are based on the synteny between the genomes of the test strain ErVv1 and the reference strain ATCC 49946 (Supplementary Figure SF2G) and on the presence of virulence genes from pathogenicity islands (a trademark genome arrangement of virulent phenotypes (Figure 4 and Supplementary Table ST9). These genomic structures might be a sign of lifestyle switching (from endophytes to pathogens and viceversa) when conditions are optimal for such phenomenon. For example, strains that have no enzymes for ascorbic acid production might be prone to take it from the environment, thus scavenging the substrates from the host's cells (Abu Kwaik and Bumann, 2013). Also, the presence of pathogenicity island components might suggest the use of such modules to achieve colonization using a pathogen-like strategy.

Schulz and colleagues proposed in 2005 that the endophytism might be an outcome of the interactions in what has been termed the “disease triangle” (i.e., the endophyte's innate virulence, the immune responses of the host, and several environmental variables). In their studies, they conclude that adaptation of endophytic fungi to plant's organs (a particular biotope) determines the neutral to beneficial association that endophytes hold with their hosts. In the light of our results, we suggest that this state of “balanced antagonism” is dictated extensively by the genomic determinants that permit such adaptation. In that sense, our results provide further evidence to reinforce this model of interactions between endophytes and plants and pave the road to further research toward the understanding of how the balanced antagonism is kept in particular hosts and how adaptation to plant may be key to the transition between pathogenic to endophytic lifestyles.

Our comparisons also show that, endophytes, epiphytes, and pathogens share a wide number of virulence-related genes. We found that core genomes are densely populated with virulence factors. These genes are present in each of the genomes in this study (Figures 2, 4) and they mark a baseline for the existence of a core virulence genome. Furthermore, the virulence-related genes found in the core genomes are conserved within each genus, regardless of pathogenic or endophytic lifestyles (Figure 4). For example, the core genome of the genus *Pantoea* is characterized by the largest number of virulence-related genes (Figure 4) as a genus-specific characteristic and genes existing in this taxon are not present in the other groups analyzed (Figure 4 and Supplementary Table ST7). This supports our hypothesis that differences between endophytes and pathogens do not exist *per se*, and demonstrates that the similarities between these two groups are set above the species level. We believe that the most abundant functions in each of the analyzed core genomes are crucial for understanding the balanced antagonism (Schulz et al., 1999) and that these functions point at the co-evolution of endophytes and pathogens. Among these, we suggest that two-component systems (especially those that regulate responses to heavy metals and those that mediate repression of gene expression), genes involved in modification of exopolysaccharide and lipopolysaccharide, genes for antibiotic resistance, efflux pumps, and cellulose metabolism are functions crucial for the endophyte-pathogenic dycotomy. These specific functions are important since they have an impact on pathogenicity as was shown for example, in genes for cellulose synthesis and catabolism (Rajeshwari et al., 2005). Previous research found that mutations in these genes can attenuate the virulent phenotype of some strains (Matthysse et al., 1995) and it is then conceivable that such mutations or downregulation of gene expression may produce an endophytic phenotype as an attenuated virulence state. We extend this rationale to other genes of the core genomes, like those coding for the phospholipase A, a well-known virulence determinant that in human pathogens is activated upon antagonistic antimicrobial activity (Istivan and Coloe, 2006); the *clpV* gene that has been linked to the proper folding of effector proteins in pathogenic strains (Schlieker et al., 2005; Filloux et al., 2008) and those virulence-related genes that are key in regulatory networks in other taxa, including the orphan *quorum sensing* gene s*diA* (Kanamaru et al., 2000) found in all the strains analyzed. Some of these functions appear more frequently in the genomes of microorganisms adopting a specific lifestyle (pathogenicity, endophytism, and other lifestyles), and their abundance may vary accross these groups with specific functions associating to specific lifestyles (Dr Pablo Hardoim, personal communication). Although our methods for calculating virulence factors may have introduced a bias (indeed this could explain why such associations are not clearly emerging from our data), the genes that we have found in this study reflect existing and previously reported functions. Together with the aformentioned findings, our observations are consistent with the emerging idea of pathobiome and “balanced antagonism,” by which host-adapted bacteria can play different roles, depending on their relations with the environment.

Complementary to the core genome, the accessory genome reveals differences in niche specialization. Variation in accessory genome size might be related to the endophytic lifestyle, since genome reduction is reported in symbiotic microorganisms (McCutcheon and Moran, 2012). Strain EnVs6 for example, displays the smallest accessory genome in the *Enterobacter* set of strains (Figure 2A), fitting the concept that endophytes have reduced genomes as compared to pathogens. In contrast, the accessory genome of the test strain ErVv1 is large, paralleling the one of the epiphyte strain Eb661 (Figure 2B). Also, genome structure in the test strains resembles that of endophytic reference genomes (Figure 2) in the number of genes that compose the accessory genome. For example, in Figure 2A the accessory genomes of strain EnVs2 and EnVs6 contain lower gene numbers, similar to what happens in the endophytic reference genomes *Enterobacter* sp. 638. The same happens in strain ErVv1, which contains about the same number of genes as the endophytic *E. billingiae* Eb661 (Figure 2B) and in strains PaVv1 and PaVv7 that are similar to the endophytic reference *P. agglomerans* 299R (Figure 2C). Niche specialization might be related to the abundance of gene functions like the toxin-antitoxin systems (Figure 4 and Supplementary Table ST5) that are thought to be more abundant in free living engaging bacteria and fewer in other types of lifestyle (Pandey and Gerdes, 2005) and the ABC-type polar amino acid transport system for opine translocation (Moore et al., 1997) that in other taxa is important for selection of bacterial pathogenic subpopulations.

Although we report several functions that are characteristically shared between endophytic test strains and reference strains with other types of lifestyles (pathogens and epiphytes), we present a set of genes that could be found only in the endophytic genomes, either test or reference strains. We are uncertain of how these genes affect virulence in endophytes. As shown in Supplementary Table ST4 the nature of this genes is quite diverse and span from functions related to the normal modification of the cell surface (in the cell wall and capsule category) to the catabolism of by-products of the S-adenosyl cystein pathway (in the regulation and cell signaling category). However, it is yet to be discovered how such modifications might be involved in the mechanisms of colonization or if they are used for beneficial associations or for pathogenicity.

Reinhold-Hurek and Hurek (2011) have highlighted the main characteristics of bacterial endophytes and the challenges for the study of this lifestyle. The term “disarmed pathogen” has arisen for endophytes that certainly hold a virulent background in their genomes while lacking a set of genes allegedly atributed to virulence. Also, these reserchers have proposed that endophytic colonization might be followed by a very mild immune response in the plant due to the presence (or absence) of microbial associated molecular patterns (MAMPs) and that these response, although related to strategies pathogens of colonization, differs in magnitud and perhaps in mechanisms. Our results agree with these points since we have been able to identify the presence of virulence factors that remind the arsenal of pathogenic bacteria (for example the presence of a collection of genes dedictated to the siderophore enterobactin synthesis in *Enterobacter* test strain, Supplementary Table ST5) but we have also shown that some of the endophytic strains lack genes that have been proven to be key for virulence in pathogenic bacteria (for example, genes involved in flagellum assembly that might be triggers of plant's immune responses, Supplementary Table ST9). We propose that some of these traits provide clues on how bacteria with an endophytic lifestyle maintain a symbiotic relation with its host as they are present sometimes only in endophytes and moreover in grape endophytes (test strains). This is the case of endophytic phage sequences (Supplementary Table ST8) which are not rare in the endophytic genomes (Ozer et al., 2014). We speculate that a link between phage sequences and the attenuation of virulence in endophytes might exist given the widespread appearance of such sequences only in the genomes of test strains. Moreover, a genome analysis of the endophytic test strains revealed that the core endophytic genome (i.e., the collection of orthologous genes present only in the endophytic test strains of our set) contains only 536 gene families (the endophytic core genome of our test strains) and is populated with functions related to vitamin synthesis and to cell signaling as well as virulence(data not shown). This suggestins that endophytic only a limited part of the genome of endophytes is dedicated to sych associations and that virulence is a leading trait in that core genome.

Summing up these observations, we suggest that endophytes conserve properties of different lifestyles, including pathogenic traits. This is reflected in the structural organization of the genomes (Figure 1) and in the overlapping functions between the test strains and the genomes of plant or animal pathogens, epiphytes, or endophytes (Figures 3, 4). We propose that endophytic and pathogenic lifestyles are composed of a base core virulence genome that might be used and expressed differentially, as has been shown for other taxa (Meysman et al., 2013). While this background virulence genome exists for all species analyzed (Figure 4, and Supplementary Table ST9), regardless of their lifestyle, there are devoted genes that permit niche specialization and occupation either in the core and in the accesory genome (Figure 2 and Table 1). This level of genomic organization makes the genomes of the test strains structurally similar to the genome of a strain that fits into one kind of lifestyle (for example strain ErVv1 being similar to the reference alignment genome strain ATCC 13047 in the genomic map of Figure 1) while functionally recalling a different lifestyle (for example genes shared between strain ErVv1 and the epiphyte Eb661 in Figure 3B). Our findings might also explain intra-genus specificity as shown for test endophytes under genera *Pantoea* and *Erwinia* that share a larger number of genes with the non-pathogenic references, while those under genus *Enterobacter* share more genes with the pathogenic reference genome (Figure 3). Lifestyle in pathogens or endophytes might be the outcome of a complex, multifactorial interaction. Our conclusions are consistent with the hypothesis that relationships between environment, host and microorganism(s) contribute to shape the environmental role of microorganisms in this symbiosis, independent of their phylogenetical relatedness. Our research is to the best of our knowledge a pioneer in two regards. First, we are showing similarities between sequenced genomes of endophytic strains from grapevine while also emphasizing on the differences that our endophytic test strains present when compared with organisms spanning other lifestyles. Secondly, we are using comparative genomics to establish a link between the genome content and genome organization of endophytic (beneficial) organisms with niche occupation, by highlighting the role of specific characteristics of the genome, that lead to different degrees of specialization.

### Conflict of interest statement

The authors declare that the research was conducted in the absence of any commercial or financial relationships that could be construed as a potential conflict of interest.

## References

[B1] Abu Kwaik Y. Bumann D. (2013). Microbial quest for food *in vivo*: “nutritional virulence” as an emerging paradigm. Cell. Microbiol. 15, 882–890. 10.1111/cmi.12138 23490329

[B2] Ahmed N. (2009). A flood of microbial genomes-do we need more? PLoS ONE 4:e5831. 10.1371/journal.pone.0005831 19513110 PMC2688083

[B3] Alexey G. Vladislav S. Nikolay V. Glenn T. (2013). QUAST: quality assessment tool for genome assemblies. Bioinformatics 29, 1072–1075. 10.1093/bioinformatics/btt086 23422339 PMC3624806

[B4] Alikhan N. F. Petty N. K. Ben Zakour N. L. Beatson S. A. (2011). BLAST Ring Image Generator (BRIG): simple prokaryote genome comparisons. BMC Genomics 12:402. 10.1186/1471-2164-12-402 21824423 PMC3163573

[B5] Amadou C. Pascal G. Mangenot S. Glew M. Bontemps C. Capela D.. (2008). Genome sequence of the beta-rhizobium *Cupriavidus taiwanensis* and comparative genomics of rhizobia. Genome Res. 18, 1472–1483. 10.1101/gr.076448.108 18490699 PMC2527706

[B6] Backert S. Schwarz T. Miehlke S. Kirsch C. Sommer C. Kwok T.. (2004). Functional analysis of the cag pathogenicity island in *Helicobacter pylori* isolates from patients with gastritis, peptic ulcer, and gastric cancer. Infect. Immun. 72, 1043–1056. 10.1128/IAI.72.2.1043-1056.2004 14742552 PMC321631

[B7] Bentley S. D. Parkhill J. (2004). Comparative genomic structure of prokaryotes. Annu. Rev. Genet. 38, 771–792. 10.1146/annurev.genet.38.072902.094318 15568993

[B8] Binnewies T. T. Motro Y. Hallin P. F. Lund O. Dunn D. La T.. (2006). Ten years of bacterial genome sequencing: comparative-genomics-based discoveries. Funct. Integr. Genomics 6, 165–185. 10.1007/s10142-006-0027-2 16773396

[B9] Bland C. Ramsey T. L. Sabree F. Lowe M. Brown K. Kyrpides N. C.. (2007). CRISPR recognition tool (CRT): a tool for automatic detection of clustered regularly interspaced palindromic repeats. BMC Bioinformatics 8:209. 10.1186/1471-2105-8-209 17577412 PMC1924867

[B10] Bordiec S. Paquis S. Lacroix H. Dhondt S. Ait Barka E. Kauffmann S.. (2011). Comparative analysis of defence responses induced by the endophytic plant growth-promoting rhizobacterium *Burkholderia phytofirmans* strain PsJN and the non-host bacterium *Pseudomonas syringae* pv. pisi in grapevine cell suspensions. J. Exp. Bot. 62, 595–603. 10.1093/jxb/erq291 20881012

[B11] Brenner D. J. Mcwhorter A. C. Kai A. Steigerwalt A. G. Farmer J. J. III. (1986). Enterobacter asburiae sp. nov., a new species found in clinical specimens, and reassignment of *Erwinia dissolvens* and *Erwinia nimipressuralis* to the genus *Enterobacter* as *Enterobacter dissolvens* comb. nov. and *Enterobacter nimipressuralis* comb. nov. J. Clin. Microbiol. 23, 1114–1120. 3711302 10.1128/jcm.23.6.1114-1120.1986 PMC268805

[B12] Britton R. A. Young V. B. (2012). Interaction between the intestinal microbiota and host in *Clostridium difficile* colonization resistance. Trends Microbiol. 20, 313–319. 10.1016/j.tim.2012.04.001 22595318 PMC3408078

[B13] Campisano A. Pancher M. Puopolo G. Puddu A. Lòpez-Fernàndez S. Biagini B. (2015). Diversity in endophytic populations reveals functional and taxonomic diversity between wild and domesticated grapevines. Am. J. Enol. Vitic. 66, 12–21. 10.5344/ajev.2014.14046

[B14] Cardenas A. Rodriguez R. L. Pizarro V. Cadavid L. F. Arevalo-Ferro C. (2012). Shifts in bacterial communities of two Caribbean reef-building coral species affected by white plague disease. ISME J. 6, 502–512. 10.1038/ismej.2011.123 21955993 PMC3280130

[B15] Cariveau D. P. Elijah Powell J. Koch H. Winfree R. Moran N. A. (2014). Variation in gut microbial communities and its association with pathogen infection in wild bumble bees (Bombus). ISME J. 8, 2369–2379. 10.1038/ismej.2014.180 24763369 PMC4260702

[B16] Charpentier E. Courvalin P. (1999). Antibiotic Resistance in *Listeria* spp. Antimicrob. Agents Chemother. 43, 2103–2108. 10471548 10.1128/aac.43.9.2103 PMC89430

[B17] Chen L. Xiong Z. Sun L. Yang J. Jin Q. (2012). VFDB 2012 update: toward the genetic diversity and molecular evolution of bacterial virulence factors. Nucleic Acids Res. 40, D641–D645. 10.1093/nar/gkr989 22067448 PMC3245122

[B18] Choi O. Lim J. Y. Seo Y.-S. Hwang I. Kim J. (2012). Complete genome sequence of the rice pathogen *Pantoea ananatis* strain PA13. J. Bacteriol. 194, 531–531. 10.1128/JB.06450-11 22207741 PMC3256675

[B19] Christensen B. B. Sternberg C. Andersen J. B. Eberl L. Møller S. Givskov M.. (1998). Establishment of new genetic traits in a microbial biofilm community. Appl. Environ. Microbiol. 64, 2247–2255. 9603843 10.1128/aem.64.6.2247-2255.1998 PMC106307

[B20] Clarke B. B. White J. F. Hurley R. H. Torres M. S. Sun S. Huff D. R. (2006). Endophyte-mediated suppression of dollar spot disease in fine fescues. Plant Dis. 90, 994–998. 10.1094/PD-90-0994 30781289

[B21] Clay K. Schardl C. (2002). Evolutionary origins and ecological consequences of endophyte symbiosis with grasses. Am. Nat. 160(Suppl. 4), S99–S127. 10.1086/342161 18707456

[B22] Compant S. Reiter B. Sessitsch A. Nowak J. Clement C. Ait Barka E. (2005). Endophytic colonization of *Vitis vinifera* L. by plant growth-promoting bacterium Burkholderia sp. strain PsJN. Appl. Environ. Microbiol. 71, 1685–1693. 10.1128/AEM.71.4.1685-1693.2005 15811990 PMC1082517

[B23] Darling A. C. Mau B. Blattner F. R. Perna N. T. (2004). Mauve: multiple alignment of conserved genomic sequence with rearrangements. Genome Res. 14, 1394–1403. 10.1101/gr.2289704 15231754 PMC442156

[B24] De Maayer P. Chan W. Y. Venter S. N. Toth I. K. Birch P. R. Joubert F.. (2010). Genome sequence of *Pantoea ananatis* LMG20103, the causative agent of *Eucalyptus* blight and dieback. J. Bacteriol. 192, 2936–2937. 10.1128/JB.00060-10 20348253 PMC2876505

[B25] Dubois T. Faegri K. Perchat S. Lemy C. Buisson C. Nielsen-Leroux C.. (2012). Necrotrophism is a quorum-sensing-regulated lifestyle in *Bacillus thuringiensis*. PLoS Pathog. 8:e1002629. 10.1371/journal.ppat.1002629 22511867 PMC3325205

[B26] Elliott S. J. Sperandio V. Giron J. A. Shin S. Mellies J. L. Wainwright L.. (2000). The locus of enterocyte effacement (LEE)-encoded regulator controls expression of both LEE- and non-LEE-encoded virulence factors in enteropathogenic and enterohemorrhagic *Escherichia coli*. Infect. Immun. 68, 6115–6126. 10.1128/IAI.68.11.6115-6126.2000 11035714 PMC97688

[B27] Falkow S. (2004). Molecular Koch's postulates applied to bacterial pathogenicity–a personal recollection 15 years later. Nat. Rev. Micro. 2, 67–72. 10.1038/nrmicro799 15035010

[B28] Filloux A. Hachani A. Bleves S. (2008). The bacterial type VI secretion machine: yet another player for protein transport across membranes. Microbiology 154, 1570–1583. 10.1099/mic.0.2008/016840-0 18524912

[B29] Fouts D. E. Tyler H. L. Deboy R. T. Daugherty S. Ren Q. Badger J. H.. (2008). Complete genome sequence of the N2-fixing broad host range endophyte *Klebsiella pneumoniae* 342 and virulence predictions verified in mice. PLoS Genet. 4:e1000141. 10.1371/journal.pgen.1000141 18654632 PMC2453333

[B30] Fujita Y. Fujita T. (1987). The gluconate operon *gnt* of *Bacillus subtilis* encodes its own transcriptional negative regulator. Proc. Natl. Acad. Sci. U.S.A. 84, 4524–4528. 10.1073/pnas.84.13.4524 3037520 PMC305122

[B31] Ghodsi M. Hill C. M. Astrovskaya I. Lin H. Sommer D. D. Koren S.. (2013). *De novo* likelihood-based measures for comparing genome assemblies. BMC Res. Notes 6:334. 10.1186/1756-0500-6-334 23965294 PMC3765854

[B32] Hall T. A. (1999). BioEdit: a user-friendly biological sequence alignment editor and analysis program for Windows 95/98/NT. Nucleic Acids Symp. Ser. 41, 95–98.

[B33] Hurek T. Handley L. L. Reinhold-Hurek B. Piche Y. (2002). *Azoarcus* grass endophytes contribute fixed nitrogen to the plant in an unculturable state. Mol. Plant-Microbe Interact. 15, 233–242. 10.1094/MPMI.2002.15.3.233 11952126

[B34] Husemann P. Stoye J. (2010). r2cat: synteny plots and comparative assembly. Bioinformatics 26, 570–571. 10.1093/bioinformatics/btp690 20015948 PMC2820676

[B35] Iniguez A. L. Dong Y. Carter H. D. Ahmer B. M. Stone J. M. Triplett E. W. (2005). Regulation of enteric endophytic bacterial colonization by plant defenses. Mol. Plant Microbe Interact. 18, 169–178. 10.1094/MPMI-18-0169 15720086

[B36] Inoue A. Murata Y. Takahashi H. Tsuji N. Fujisaki S. Kato J.-I. (2008). Involvement of an essential gene, *mviN*, in murein synthesis in *Escherichia coli*. J. Bacteriol. 190, 7298–7301. 10.1128/JB.00551-08 18708495 PMC2580715

[B37] Ionescu M. Franchini A. Egli T. Belkin S. (2008). Induction of the *yjbEFGH* operon is regulated by growth rate and oxygen concentration. Arch. Microbiol. 189, 219–226. 10.1007/s00203-007-0311-0 17957352

[B38] Istivan T. S. Coloe P. J. (2006). Phospholipase A in Gram-negative bacteria and its role in pathogenesis. Microbiology 152, 1263–1274. 10.1099/mic.0.28609-0 16622044

[B39] Iuchi S. Matsuda Z. Fujiwara T. Lin E. C. (1990). The *arcB* gene of *Escherichia coli* encodes a sensor-regulator protein for anaerobic repression of the *arc* modulon. Mol. Microbiol. 4, 715–727. 10.1111/j.1365-2958.1990.tb00642.x 2201868

[B40] Kahlke T. Goesmann A. Hjerde E. Willassen N. P. Haugen P. (2012). Unique core genomes of the bacterial family vibrionaceae: insights into niche adaptation and speciation. BMC Genomics 13:179. 10.1186/1471-2164-13-179 22574681 PMC3464603

[B41] Kanamaru K. Kanamaru K. Tatsuno I. Tobe T. Sasakawa C. (2000). SdiA, an *Escherichia coli* homologue of quorum-sensing regulators, controls the expression of virulence factors in enterohaemorrhagic *Escherichia coli* O157:H7. Mol. Microbiol. 38, 805–816. 10.1046/j.1365-2958.2000.02171.x 11115115

[B42] Kelly B. G. Vespermann A. Bolton D. J. (2009). Horizontal gene transfer of virulence determinants in selected bacterial foodborne pathogens. Food Chem. Toxicol. 47, 969–977. 10.1016/j.fct.2008.02.007 18420327

[B43] Kube M. Migdoll A. M. Gehring I. Heitmann K. Mayer Y. Kuhl H.. (2010). Genome comparison of the epiphytic bacteria *Erwinia billingiae* and *E. tasmaniensis* with the pear pathogen *E. pyrifoliae*. BMC Genomics 11:393. 10.1186/1471-2164-11-393 20565991 PMC2897811

[B44] Li L. Stoeckert C. J. Jr. Roos D. S. (2003). OrthoMCL: identification of ortholog groups for eukaryotic genomes. Genome Res. 13, 2178–2189. 10.1101/gr.1224503 12952885 PMC403725

[B45] Li X. Hu Y. Gong J. Zhang L. Wang G. (2013). Comparative genome characterization of *Achromobacter* members reveals potential genetic determinants facilitating the adaptation to a pathogenic lifestyle. Appl. Microbiol. Biotechnol. 97, 6413–6425. 10.1007/s00253-013-5018-3 23749121

[B46] Lindquist S. Weston-Hafer K. Schmidt H. Pul C. Korfmann G. Erickson J.. (1993). AmpG, a signal transducer in chromosomal beta-lactamase induction. Mol. Microbiol. 9, 703–715. 10.1111/j.1365-2958.1993.tb01731.x 8231804

[B47] Linhartová I. Bumba L. Mašín J. Basler M. Osička R. Kamanová J.. (2010). RTX proteins: a highly diverse family secreted by a common mechanism. FEMS Microbiol. Rev. 34, 1076–1112. 10.1111/j.1574-6976.2010.00231.x 20528947 PMC3034196

[B48] Little A. E. Robinson C. J. Peterson S. B. Raffa K. F. Handelsman J. (2008). Rules of engagement: interspecies interactions that regulate microbial communities. Annu. Rev. Microbiol. 62, 375–401. 10.1146/annurev.micro.030608.101423 18544040

[B49] Lomovskaya O. Lewis K. (1992). *emr*, an *Escherichia coli* locus for multidrug resistance. Proc. Natl. Acad. Sci. U.S.A. 89, 8938–8942. 10.1073/pnas.89.19.8938 1409590 PMC50039

[B50] Luo R. Liu B. Xie Y. Li Z. Huang W. Yuan J.. (2012). SOAPdenovo2: an empirically improved memory-efficient short-read *de novo* assembler. Gigascience 1:18. 10.1186/2047-217X-1-18 23587118 PMC3626529

[B51] Maddocks S. E. Oyston P. C. (2008). Structure and function of the LysR-type transcriptional regulator (LTTR) family proteins. Microbiology 154, 3609–3623. 10.1099/mic.0.2008/022772-0 19047729

[B53] Matthysse A. G. White S. Lightfoot R. (1995). Genes required for cellulose synthesis in Agrobacterium tumefaciens. J. Bacteriol. 177, 1069–1075. 7860585 10.1128/jb.177.4.1069-1075.1995 PMC176703

[B54] McCandlish A. C. Silhavy T. J. (2007). Sugar-coating bacteria with lipopolysaccharides. Microbe 2, 289 10.1128/microbe.2.289.1

[B55] McCutcheon J. P. Moran N. A. (2012). Extreme genome reduction in symbiotic bacteria. Nat. Rev. Microbiol. 10, 13–26. 10.1038/nrmicro2670 22064560

[B56] Meng Y. Li Y. Galvani C. D. Hao G. Turner J. N. Burr T. J.. (2005). Upstream migration of *Xylella fastidiosa* via pilus-driven twitching motility. J. Bacteriol. 187, 5560–5567. 10.1128/JB.187.16.5560-5567.2005 16077100 PMC1196070

[B57] Meysman P. Sanchez-Rodriguez A. Fu Q. Marchal K. Engelen K. (2013). Expression divergence between *Escherichia coli* and *Salmonella enterica* serovar Typhimurium reflects their lifestyles. Mol. Biol. Evol. 30, 1302–1314. 10.1093/molbev/mst029 23427276 PMC3649669

[B58] Mitter B. Petric A. Shin M. W. Chain P. S. G. Hauberg-Lotte L. Reinhold-Hurek B.. (2013). Comparative genome analysis of *Burkholderia phytofirmans* PsJN reveals a wide spectrum of endophytic lifestyles based on interaction strategies with host plants. Front. Plant. Sci. 4:120. 10.3389/fpls.2013.00120 23641251 PMC3639386

[B59] Monack D. M. Mueller A. Falkow S. (2004). Persistent bacterial infections: the interface of the pathogen and the host immune system. Nat. Rev. Microbiol. 2, 747–765. 10.1038/nrmicro955 15372085

[B60] Moore L. W. Chilton W. S. Canfield M. L. (1997). Diversity of opines and opine-catabolizing bacteria isolated from naturally occurring crown gall tumors. Appl. Environ. Microbiol. 63, 201–207. 16535484 10.1128/aem.63.1.201-207.1997 PMC1389099

[B61] Munkelt D. Grass G. Nies D. H. (2004). The chromosomally encoded cation diffusion facilitator proteins DmeF and FieF from *Wautersia metallidurans* CH34 are transporters of broad metal specificity. J. Bacteriol. 186, 8036–8043. 10.1128/JB.186.23.8036-8043.2004 15547276 PMC529076

[B62] Oh C. S. Kim J. F. Beer S. V. (2005). The Hrp pathogenicity island of *Erwinia amylovora* and identification of three novel genes required for systemic infectiondouble dagger. Mol. Plant. Pathol. 6, 125–138. 10.1111/j.1364-3703.2005.00269.x 20565644

[B63] Ozer E. A. Allen J. P. Hauser A. R. (2014). Characterization of the core and accessory genomes of *Pseudomonas aeruginosa* using bioinformatic tools Spine and AGEnt. BMC Genomics 15:737. 10.1186/1471-2164-15-737 25168460 PMC4155085

[B64] Pandey D. P. Gerdes K. (2005). Toxin-antitoxin loci are highly abundant in free-living but lost from host-associated prokaryotes. Nucleic Acids Res. 33, 966–976. 10.1093/nar/gki201 15718296 PMC549392

[B65] Park D. H. Thapa S. P. Choi B. S. Kim W. S. Hur J. H. Cho J. M.. (2011). Complete genome sequence of Japanese *Erwinia* strain ejp617, a bacterial shoot blight pathogen of pear. J. Bacteriol. 193, 586–587. 10.1128/JB.01246-10 21075933 PMC3019831

[B66] Parker C. T. Sperandio V. (2009). Cell-to-cell signalling during pathogenesis. Cell. Microbiol. 11, 363–369. 10.1111/j.1462-5822.2008.01272.x 19068097 PMC2786497

[B67] Parkhill J. Sebaihia M. Preston A. Murphy L. D. Thomson N. Harris D. E.. (2003). Comparative analysis of the genome sequences of *Bordetella pertussis*, *Bordetella parapertussis* and *Bordetella bronchiseptica*. Nat. Genet. 35, 32–40. 10.1038/ng1227 12910271

[B68] Parsons J. F. Song F. Parsons L. Calabrese K. Eisenstein E. Ladner J. E. (2004). Structure and function of the phenazine biosynthesis protein PhzF from *Pseudomonas fluorescens* 2-79. Biochemistry 43, 12427–12435. 10.1021/bi049059z 15449932

[B69] Poole K. Braun V. (1988). Iron regulation of *Serratia marcescens* hemolysin gene expression. Infect. Immun. 56, 2967–2971. 3049376 10.1128/iai.56.11.2967-2971.1988 PMC259678

[B70] Rajeshwari R. Jha G. Sonti R. V. (2005). Role of an in planta-expressed xylanase of *Xanthomonas oryzae* pv. *oryzae* in promoting virulence on rice. Mol. Plant-Microbe Interact. 18, 830–837. 10.1094/MPMI-18-0830 16134895

[B71] R Core Team. (2013). R: A Language and Environment for Statistical Computing. Vienna:R Foundation for Statistical Computing Available online at: http://www.R-project.org/.

[B72] Reinhold-Hurek B. Hurek T. (2011). Living inside plants: bacterial endophytes. Curr. Opin. Plant. Biol. 14, 435–443. 10.1016/j.pbi.2011.04.004 21536480

[B73] Remus-Emsermann M. N. Kim E. B. Marco M. L. Tecon R. Leveau J. H. (2013). Draft genome sequence of the phyllosphere model bacterium *Pantoea agglomerans* 299R. Genome announc. 1, e00036–e00013. 10.1128/genomeA.00036-13 23472227 PMC3587929

[B74] Ren Y. Ren Y. Zhou Z. Guo X. Li Y. Feng L.. (2010). Complete genome sequence of *Enterobacter cloacae* subsp. *cloacae* type strain ATCC 13047. J. Bacteriol. 192, 2463–2464. 10.1128/JB.00067-10 20207761 PMC2863489

[B75] Rosas-Magallanes V. Deschavanne P. Quintana-Murci L. Brosch R. Gicquel B. Neyrolles O. (2006). Horizontal transfer of a virulence operon to the ancestor of *Mycobacterium tuberculosis*. Mol. Biol. Evol. 23, 1129–1135. 10.1093/molbev/msj120 16520338

[B76] Sakai D. Komano T. (2000). The *pilL* and *pilN* genes of IncI1 plasmids R64 and ColIb-P9 encode outer membrane lipoproteins responsible for thin pilus biogenesis. Plasmid 43, 149–152. 10.1006/plas.1999.1434 10686134

[B77] Sana T. G. Soscia C. Tonglet C. M. Garvis S. Bleves S. (2013). Divergent control of two type VI secretion systems by RpoN in *Pseudomonas aeruginosa*. PLoS ONE 8:e76030. 10.1371/journal.pone.0076030 24204589 PMC3804575

[B78] Schlieker C. Zentgraf H. Dersch P. Mogk A. (2005). ClpV, a unique Hsp100/Clp member of pathogenic proteobacteria. Biol. Chem. 386, 1115–1127. 10.1515/BC.2005.128 16307477

[B79] Schulz B. Boyle C. (2005). The endophytic continuum. Mycol. Res. 109, 661–686. 10.1017/S095375620500273X 16080390

[B80] Schulz B. Römmert A.-K. Dammann U. Aust H.-J. Strack D. (1999). The endophyte-host interaction: a balanced antagonism? Mycol. Res. 103, 1275–1283. 10.1017/S0953756299008540

[B81] Sebaihia M. Bocsanczy A. Biehl B. Quail M. Perna N. Glasner J.. (2010). Complete genome sequence of the plant pathogen *Erwinia amylovora* strain ATCC 49946. J. Bacteriol. 192, 2020–2021. 10.1128/JB.00022-10 20118253 PMC2838050

[B82] Seemann T. (2014). Prokka: rapid prokaryotic genome annotation. Bioinformatics 30, 2068–2069. 10.1093/bioinformatics/btu153 24642063

[B83] Shankar V. Hamilton M. J. Khoruts A. Kilburn A. Unno T. Paliy O.. (2014). Species and genus level resolution analysis of gut microbiota in *Clostridium difficile* patients following fecal microbiota transplantation. Microbiome 2:13. 10.1186/2049-2618-2-13 24855561 PMC4030581

[B84] Shepard W. Soutourina O. Courtois E. England P. Haouz A. Martin-Verstraete I. (2011). Insights into the Rrf2 repressor family–the structure of CymR, the global cysteine regulator of *Bacillus subtilis*. FEBS J. 278, 2689–2701. 10.1111/j.1742-4658.2011.08195.x 21624051

[B85] Shiba Y. Matsumoto K. Hara H. (2006). DjlA negatively regulates the Rcs signal transduction system in *Escherichia coli*. Genes Genet. Syst. 81, 51–56. 10.1266/ggs.81.51 16607041

[B86] Shimada T. Fujita N. Yamamoto K. Ishihama A. (2011). Novel Roles of cAMP Receptor Protein (CRP) in regulation of transport and metabolism of carbon sources. PLoS ONE 6:e20081. 10.1371/journal.pone.0020081 21673794 PMC3105977

[B87] Shin S. H. Kim S. Kim J. Y. Lee S. Um Y. Oh M. K.. (2012). Complete genome sequence of *Enterobacter aerogenes* KCTC 2190. J. Bacteriol. 194, 2373–2374. 10.1128/JB.00028-12 22493190 PMC3347075

[B88] Smits T. H. Rezzonico F. Kamber T. Goesmann A. Ishimaru C. A. Stockwell V. O.. (2010). Genome sequence of the biocontrol agent *Pantoea vagans* strain C9-1. J. Bacteriol. 192, 6486–6487. 10.1128/JB.01122-10 20952567 PMC3008540

[B89] Sorensen K. I. Hove-Jensen B. (1996). Ribose catabolism of *Escherichia coli*: characterization of the *rpiB* gene encoding ribose phosphate isomerase B and of the *rpiR* gene, which is involved in regulation of *rpiB* expression. J. Bacteriol. 178, 1003–1011. 8576032 10.1128/jb.178.4.1003-1011.1996 PMC177759

[B90] Suen G. Scott J. J. Aylward F. O. Adams S. M. Tringe S. G. Pinto-Tomás A. A.. (2010). An insect herbivore microbiome with high plant biomass-degrading capacity. PLoS Genet. 6:e1001129. 10.1371/journal.pgen.1001129 20885794 PMC2944797

[B91] Sugawara M. Epstein B. Badgley B. D. Unno T. Xu L. Reese J.. (2013). Comparative genomics of the core and accessory genomes of 48 *Sinorhizobium* strains comprising five genospecies. Genome Biol. 14:R17. 10.1186/gb-2013-14-2-r17 23425606 PMC4053727

[B92] Taghavi S. van der Lelie D. Hoffman A. Zhang Y.-B. Walla M. D. Vangronsveld J.. (2010). Genome sequence of the plant growth promoting endophytic bacterium *Enterobacter* sp. 638. PLoS Genet. 6:e1000943. 10.1371/journal.pgen.1000943 20485560 PMC2869309

[B93] Tamura K. Stecher G. Peterson D. Filipski A. Kumar S. (2013). MEGA6: molecular evolutionary genetics analysis version 6.0. Mol. Biol. Evol. 30, 2725–2729. 10.1093/molbev/mst197 24132122 PMC3840312

[B94] Tian C. Zhou Y. Zhang Y. Li Q. Zhang Y. Li D.. (2012). Comparative genomics of rhizobia nodulating soybean suggests extensive recruitment of lineage-specific genes in adaptations. Proc. Natl. Acad. Sci. U.S.A. 109, 8629–8634. 10.1073/pnas.1120436109 22586130 PMC3365164

[B95] Tritt A. Eisen J. A. Facciotti M. T. Darling A. E. (2012). An integrated pipeline for *de Novo* assembly of microbial genomes. PLoS ONE 7:e42304. 10.1371/journal.pone.0042304 23028432 PMC3441570

[B96] Troxler J. Azelvandre P. Zala M. Defago G. Haas D. (1997). Conjugative transfer of chromosomal genes between fluorescent pseudomonads in the rhizosphere of wheat. Appl. Environ. Microbiol. 63, 213–219. 16535486 10.1128/aem.63.1.213-219.1997 PMC1389100

[B97] Ur Rahman S. Van Ulsen P. (2013). System specificity of the TpsB transporters of coexpressed two-partner secretion systems of *Neisseria meningitidis*. J. Bacteriol. 195, 788–797. 10.1128/JB.01355-12 23222722 PMC3562106

[B98] Vayssier-Taussat M. Albina E. Citti C. Cosson J. F. Jacques M.-A. Lebrun M.-H.. (2014). Shifting the paradigm from pathogens to pathobiome: new concepts in the light of meta-omics. Front. Cell. Infect. Microbiol. 4:29. 10.3389/fcimb.2014.00029 24634890 PMC3942874

[B99] Wall L. Christiansen T. Orwant J. (2000). Programming Perl. California, CA: O'Reilly and Associates, Inc.

[B100] Wheeler D. L. Barrett T. Benson D. A. Bryant S. H. Canese K. Chetvernin V.. (2007). Database resources of the national center for biotechnology information. Nucleic Acids Res. 35, D5–D12. 10.1093/nar/gkm1000 17170002 PMC1781113

[B101] Xu X.-H. Su Z.-Z. Wang C. Kubicek C. P. Feng X.-X. Mao L.-J.. (2014). The rice endophyte *Harpophora oryzae* genome reveals evolution from a pathogen to a mutualistic endophyte. Sci. Rep. 4:5783. 10.1038/srep05783 25048173 PMC4105740

[B102] Yan Y. Yang J. Dou Y. Chen M. Ping S. Peng J.. (2008). Nitrogen fixation island and rhizosphere competence traits in the genome of root-associated *Pseudomonas stutzeri* A1501. Proc. Natl. Acad. Sci. U.S.A. 105:5783 7564–7569. 10.1073/pnas.0801093105 18495935 PMC2396677

[B103a] Zerbino D. R. Birney E. (2008). Velvet: algorithms for de novo short read assembly using de Bruijn graphs. Genome. Res. 18, 821–829. 10.1101/gr.074492.107 18349386 PMC2336801

[B103] Zhou C. E. Smith J. Lam M. Zemla A. Dyer M. D. Slezak T. (2007). MvirDB–a microbial database of protein toxins, virulence factors and antibiotic resistance genes for bio-defence applications. Nucleic Acids Res. 35, D391–D394. 10.1093/nar/gkl791 17090593 PMC1669772

[B104] Zhou Y. Liang Y. Lynch K. H. Dennis J. J. Wishart D. S. (2011). PHAST: A Fast Phage Search Tool. Nucleic Acids Res. 39, W347–W352. 10.1093/nar/gkr485 21672955 PMC3125810

[B105] Zuccaro A. Lahrmann U. Güldener U. Langen G. Pfiffi S. Biedenkopf D.. (2011). Endophytic life strategies decoded by genome and transcriptome analyses of the mutualistic root symbiont *Piriformospora indica*. PLoS Pathog. 7:e1002290. 10.1371/journal.ppat.1002290 22022265 PMC3192844

