# Supplementary figures and images for: Whole-genome comparative analysis of virulence genes unveils similarities and differences between endophytes and other symbiotic bacteria
Source: Front Microbiol. 2015 May 26;6:419. doi: 10.3389/fmicb.2015.00419 (PMC4443252; doi:10.3389/fmicb.2015.00419)

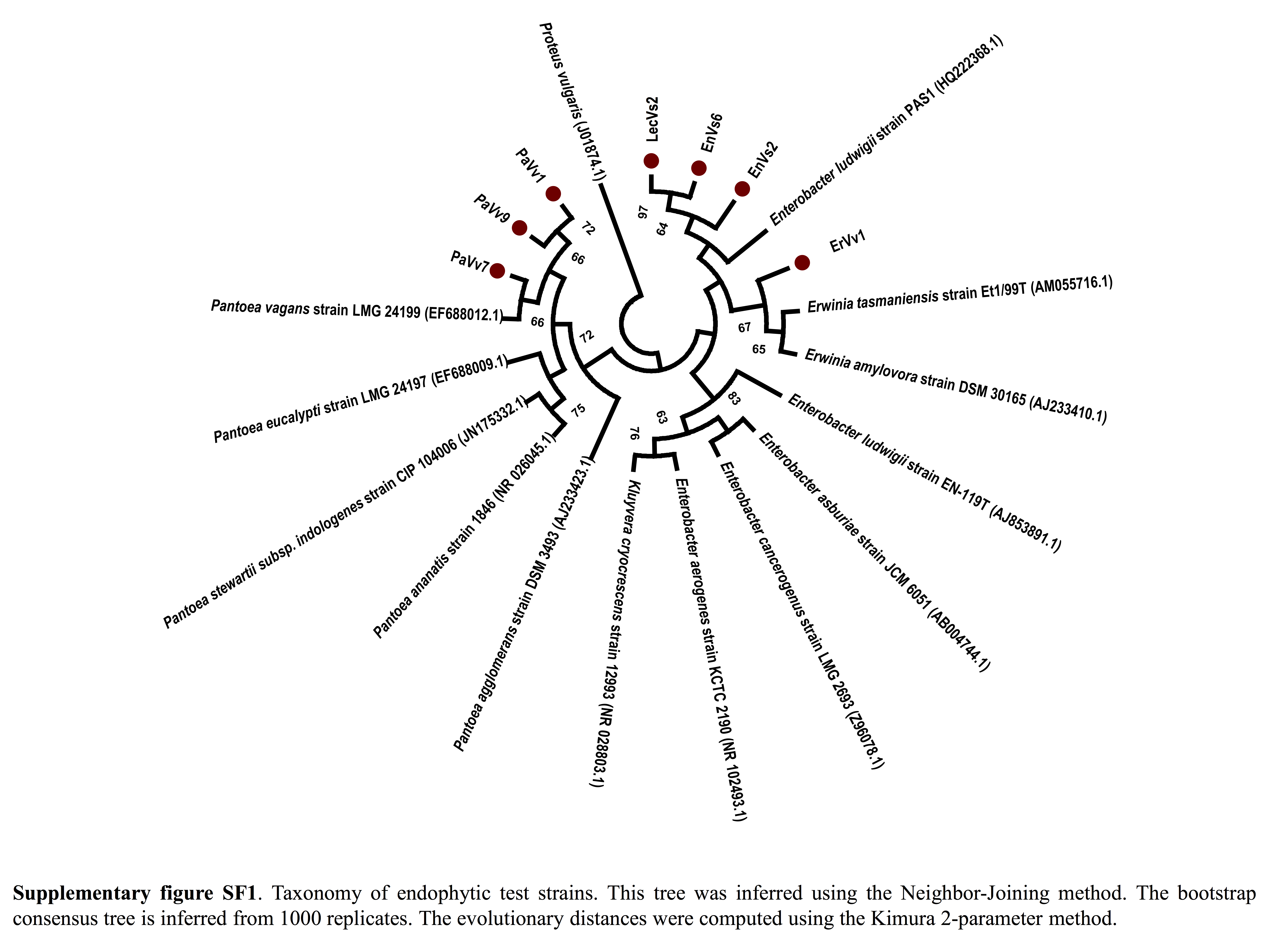

Supplement: Supplementary file 1 [file Image1.TIFF]

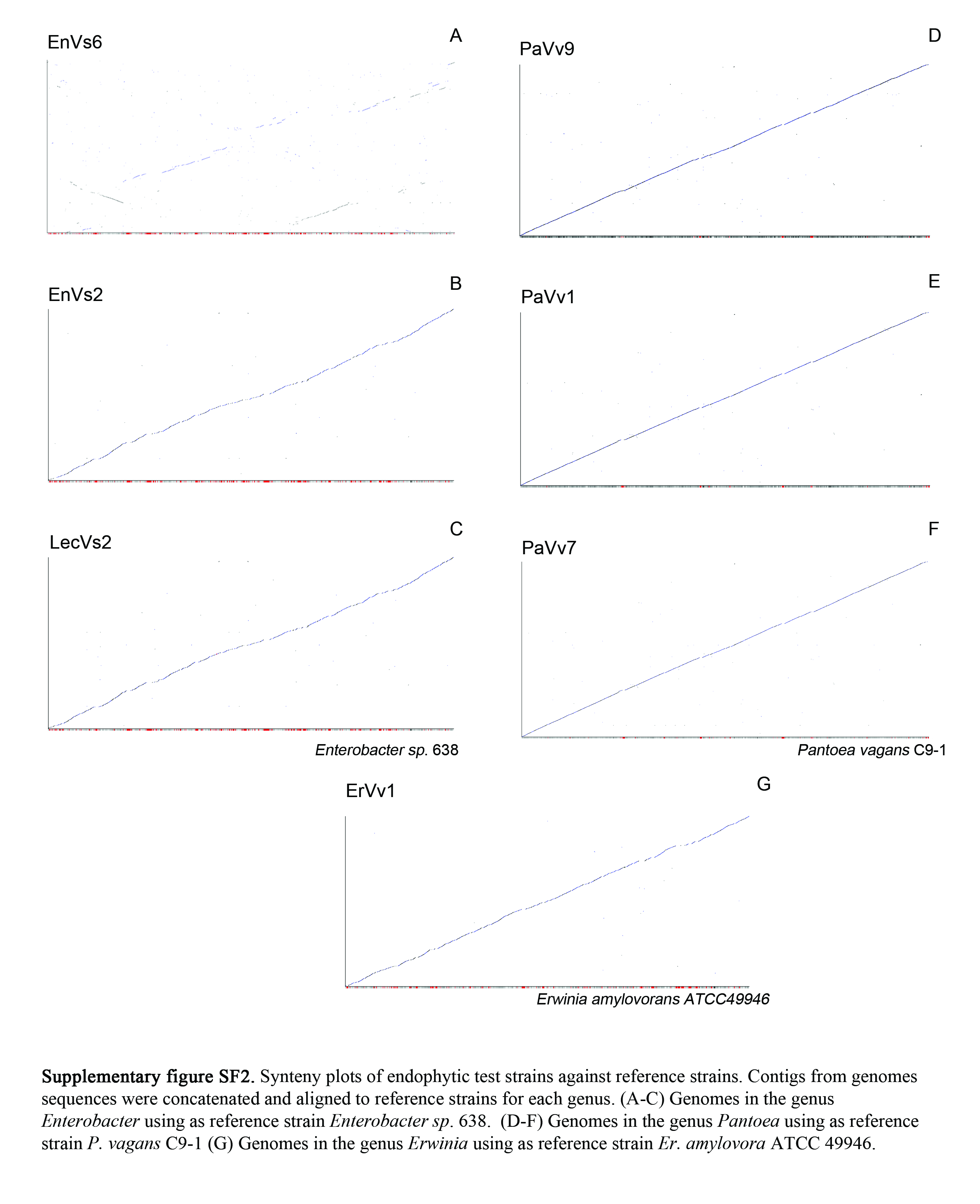

Supplement: Supplementary file 2 [file Image2.TIF]

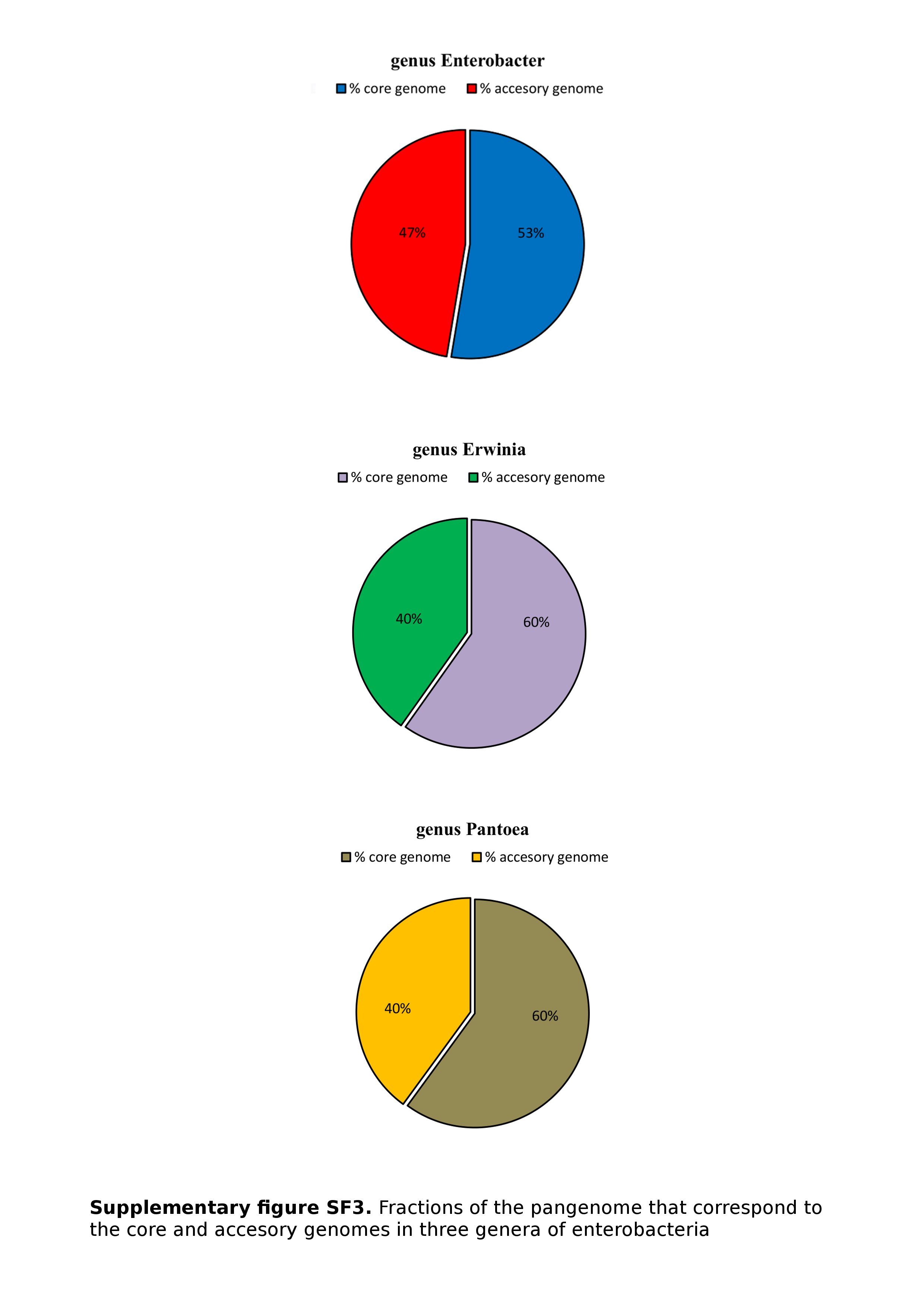

Supplement: Supplementary file 3 [file Image3.TIFF]
